# Primary solitary plasmacytoma of the liver – successful treatment with fractionated stereotactic radiotherapy (Cyberknife®): a case report

**DOI:** 10.1186/s13256-017-1358-4

**Published:** 2017-07-18

**Authors:** Thomas Chalopin, Isabelle Barillot, Jean-Paul Biny, Flavie Arbion, Marie Besson, Maria Santiago-Ribeiro, Eric Piver, Olivier Herault, Emmanuel Gyan, Lotfi Benboubker

**Affiliations:** 1 Department of Hematology, University Hospital of Tours, Tours, France; 2 Department of Radiation Oncology (CORAD), University Hospital of Tours, Tours, France; 3 Anatomopathology Center of Origet, Saint-Cyr sur Loire, France; 4 Department of Anatomopathology, University Hospital of Tours, Tours, France; 5 Department of Medical Imaging, University Hospital of Tours, Tours, France; 6 Department of Nuclear Medicine, University Hospital of Tours, Tours, France; 7 Department of Biochemistry, University Hospital of Tours, Tours, France; 8 Department of Biologic Hematology, University of Tours, Tours, France; 9 Centre Hospitalier Universitaire de Tours, Hôpital Bretonneau, 2 boulevard Tonnellé, 37044 Tours cedex 9, France

**Keywords:** Solitary plasmacytoma, Liver, Radiosurgery

## Abstract

**Background:**

Solitary plasmacytoma of the liver is a very rare and aggressive form of plasma cell dyscrasia. To the best of our knowledge, very few cases have been reported without systemic disease. We reported a rare case of hepatic solitary plasmacytoma that successfully responded to fractionated stereotactic radiotherapy.

**Case presentation:**

A 64-year-old white French man had monoclonal gammopathy of the immune globulin G lambda type; he developed a cholestasis and cytolysis with the discovery of a subscapular nodule. A biopsy showed plasma cells and, for several reasons, the decision was made to use the fractionated stereotactic radiotherapy strategy. After 20 months, he is asymptomatic and the immune globulin G component has completely disappeared.

**Conclusion:**

We suggest considering Cyberknife® radiosurgery as an option for the treatment of hepatic solitary plasmacytoma.

## Background

Solitary plasmacytoma (SP) is a rare form of plasma cell dyscrasia with a single bone (SBP) or extramedullary location (EMP). It accounts for 3 to 5% of all plasma cell neoplasms [1]. Almost 85% of EMPs involve the head and neck mucosa, especially in the upper respiratory tract [2]. Gastrointestinal, lung, bladder, and testis involvement have also been reported. Only a few cases of hepatic SP have been reported to date [3, 4]. The presence of plasma cells in the liver or spleen is known to be associated with a more aggressive form of multiple myeloma [3] that usually requires a heavy treatment with chemotherapy or consolidation therapy with autologous hematopoietic stem cell transplantation.

Radiotherapy is the treatment of choice for EMP, with excellent response rates of approximately 86% [5]. Fractionated stereotactic radiotherapy has been used for approximately two decades to deliver ablative high-dose irradiations to small target volumes and low-dose irradiations to adjacent normal tissue. It plays an increasingly important role in the therapeutic arsenal of primary and secondary liver tumors but, to the best of our knowledge, its use has never been described in the treatment of hepatic plasmacytoma. We present a rare case of hepatic SP that successfully responded to a treatment with Cyberknife®.

## Case presentation

**Fig. 1:**
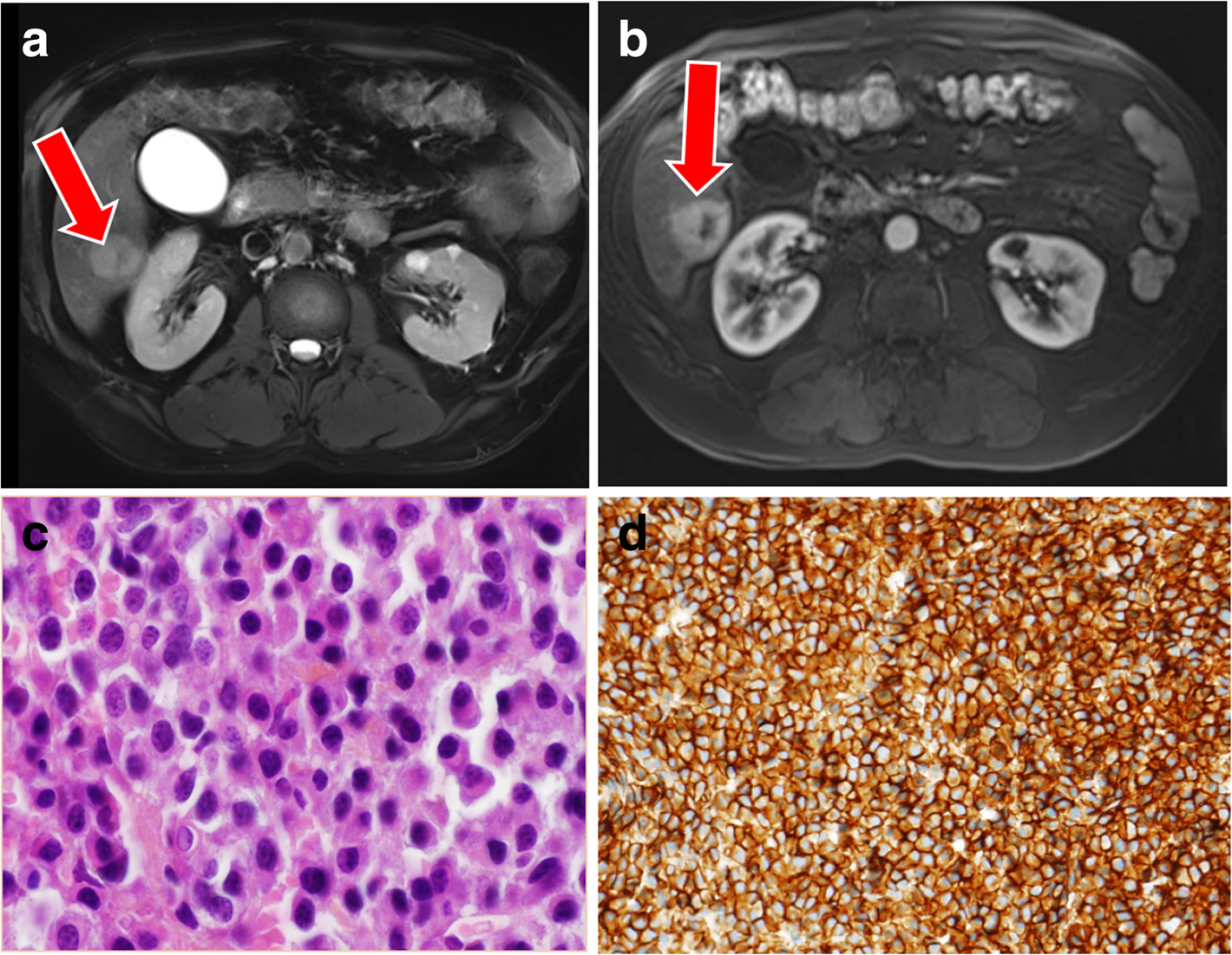
Magnetic resonance imaging images and tissue sections at diagnosis. **a** The lesion is hyperintense on the T2-weighted image (*arrow*) **b** and on the T1-weighted image with arterial phase enhancement with a centered hypointense signal in the probably necrotic tissue (*arrow*). **c** Diffuse proliferation of small monotonous round-to-ovoid cells with eccentric cytoplasms and round nuclei with **d** immunohistochemical analysis showing CD138 staining

A 64-year-old white French man had stable monoclonal gammopathy of the immune globulin G (IgG) lambda type (IgG = 1 g/L) with normal bone X-rays. He had no other significant comorbidities, except for diabetes and hypertension. Two years after the diagnosis of monoclonal gammopathy, he developed cholestasis 10N (10 x normal level) and cytolysis 4N (4 x normal value). Ultrasounds revealed a 4-cm subcapsular nodule involving segment VI of his liver. Magnetic resonance imaging (MRI) revealed multiple signal anomalies, with hemangiomas and benign cysts and a slightly hyperintense nodule on the T2-weighted image (Fig. 1a) and T1-weighted image, with arterial phase enhancement (Fig. 1b). Positron emission tomography-computed tomography (PET-CT) with ^18^fluorodeoxyglucose (^18^FDG) revealed an unusual hypermetabolic nodule, with maximum standardized uptake value (SUV_max_) at 16. An ultrasound-guided biopsy showed numerous plasma cells (Fig. 1c) with a strong CD138 staining and low lambda light chain intensity (Fig. 1d). A full blood count revealed: hemoglobin 140 g/L, platelets 160 G/L, and leukocytes 5 G/L. All other laboratory parameters including renal function, albumin, beta-2-microglobulin, and calcium were within the normal range. A bone marrow examination and skeletal assessment were normal and a diagnosis of hepatic EMP was made. Surgery was deemed too difficult to perform because of the proximity of the kidney, and standard conformal irradiation was also contraindicated for the same reason and because the lesion was highly mobile due to breathing movements. However, our multidisciplinary team suggested the use of fractionated stereotactic radiotherapy in synchrony mode using implanted fiducials to limit liver toxicity in this patient with a single lesion without bone marrow involvement.

**Fig. 2:**
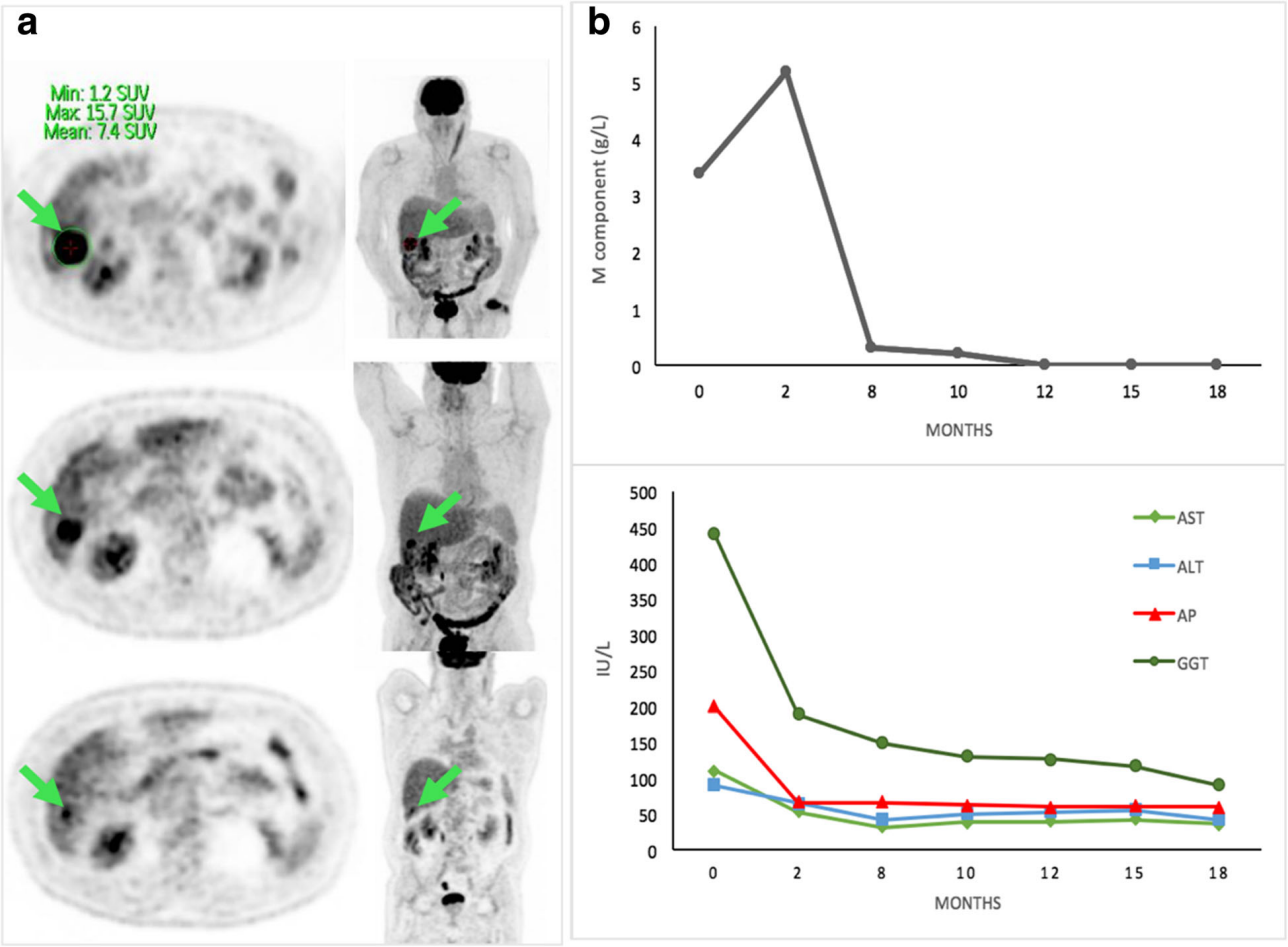
^18^Fluorodeoxyglucose positron emission tomography-computed tomography images and biologic graphics prior to and following therapy. **a** Images showing axial and coronal ^18^fluorodeoxyglucose positron emission tomography-computed tomography of the lesion in segment VI before radiosurgery, at month 12 with a 50% decrease and at month 18 with a 75% decrease. The *arrow* indicates the plasmacytoma lesion in both planes. All images show a partial remission of the metabolic activity of the nodule after Cyberknife® therapy compared to month 0. **b** The figures show reduced IgG M component with a maximum of 5.2 g of IgG/L and standardization from month 12 with normal immunofixation. The graphic *below* shows the concentration of liver enzymes, alkaline phosphatase, and gamma-glutamyltransferase. *ALT* alanine aminotransferase, *AP* alkaline phosphatase, *AST* aspartate aminotransferase, *GGT* gamma-glutamyltransferase, *SUV* standardized uptake value

In February 2015, our patient received 30 Gy in five consecutive fractions of 6 Gy, a dose that may be considered biologically equivalent to 40 Gy delivered in 2 Gy per fraction. The tolerance was excellent without acute toxicity. At month 3, ^18^FDG PET-CT revealed a stable disease, and subsequent assessments showed a partial response of 50% and 75% at month 12 and month 18, respectively (Fig. 2a). SUV_max_ also decreased to 8.2 at month 12 and 4.8 at month 18. Twenty months after irradiation, he is still asymptomatic with a normal biological liver function and IgG lambda M component has completely disappeared (Fig. 2b). No significant toxicity was observed during and after radiosurgery.

## Discussion

We describe a very good partial response of a very rare form of hepatic EMP with fractionated stereotactic radiotherapy. The diagnosis was made according to the recently updated recommendations [6], and differential diagnoses such as other neoplasms [7] were ruled out by histologic examination.

Radiation therapy is the best option for patients with localized EMP over systemic chemotherapy. Recommendations include a radiotherapy dose of 40 Gy in 20 fractions for tumors <5 cm and up to 50 Gy in 25 fractions for tumors ≥5 cm with at least a 2-cm margin encompassing the primary tumor [8]. Other alternatives exist like surgery or combined chemotherapy treatment, but are not justified outside clinical trials. Hepatic plasmacytomas without systemic disease have been treated either by chemotherapy (melphalan/steroids or vincristine, adriamycin, and dexamethasone combinations) or by radiotherapy [9]. Only a few cases of SP treated by fractionated stereotactic radiotherapy have been reported, and our case is the first reported with a hepatic localization. Wong *et al*. reported a plasmacytoma of the clivus extending to the foramen magnum treated by fractionated stereotactic radiotherapy with a complete and durable response on MRI at 12 months [10]. Theiler *et al*. reported the case of a 49-year-old man with SP of the bone, with radiotherapy as first-line treatment and radiosurgery for the treatment of local relapse with disappearance of bone lesion [11]. Other cases reported Cyberknife® success in plasmacytoma in neurological localizations such as cavernous sinus [12], cranial [13], and spine cord [14]. To the best of our knowledge, this is the first case of successful fractionated stereotactic radiotherapy use in hepatic SP. The absence of toxicity observed in this unusual observation suggests that other patients with similar presentations could benefit from this treatment modality.

## Conclusions

This case shows that stereotactic radiotherapy could be highly effective in localized liver plasmacytoma. The Cyberknife® technology, which combines high precision and surrounding tissue protection, could be an alternative to conventional radiotherapy in some cases [11, 12]. Due to the lack of guidelines and clinical trials, the indication of fractionated stereotactic radiotherapy for the treatment of plasmacytoma should be further studied. In our case, the radiographic response was almost complete, safety was satisfactory, and our patient is still asymptomatic after 20 months. We suggest considering fractionated stereotactic radiotherapy as an option for the treatment of hepatic SP.

## References

[1] Dimopoulos MA, Hamilos G. Solitary bone plasmacytoma and extramedullary plasmacytoma. Curr Treat Options Oncol. 2002;3:255–9. 10.1007/s11864-002-0015-2 12057071

[2] Finsinger P, Grammatico S, Chisini M, Piciocchi A, Foa R, Petrucci MT. Clinical features and prognostic factors in solitary plasmacytoma. Br J Haematol. 2016;172(4):554–60. 10.1111/bjh.13870 26684545

[3] Petrucci MT, Tirindelli MC, De Muro M, Levi A, Mandelli F. Extramedullary Liver Plasmacytoma: a rare presentation. Leuk Lymphoma. 2003;44(6):1075–6. 10.1080/1042819031000067882 12854914

[4] Husaric S, Pasic J, Alic E, Kuljanin M. Solitary extramedullary plasmacytoma of the liver. Acta Med Acad. 2013;42(1):85–6. 10.5644/ama2006-124.76 23735072

[5] Knobel D, Zouhair A, Tsang RW, Poortmans P, Belkacemi Y, Bolla M, Oner FD, Landmann C, Castelain B, Ozsahin M. Prognostic factors in solitary plasmacytoma of the bone: a multicenter Rare Cancer Network study. BMC Cancer. 2006;6:118. 10.1186/1471-2407-6-118 PMC1479355 16677383

[6] Rajkumar SV, Dimopoulos MA, Palumbo A, Blade J, Merlini G, Mateos MV, Miguel JFS. International Myeloma Working Group updated criteria for the diagnosis of multiple myeloma. Lancet Oncol. 2014;15(12):e538–48. 10.1016/S1470-2045(14)70442-5 25439696

[7] Ng P, Slater S, Radvan G, Price A. Hepatic plasmacytomas : Case report and review of imaging features. Australas Radiol. 1999;43(1):98–101. 10.1046/j.1440-1673.1999.00603.x 10901880

[8] Soutar R, Lucraft H, Jackson G, Reece A, Bird J, Low E, Samson D. Guidelines on the diagnosis and management of solitary plasmacytoma of bone and solitary extramedullary plasmacytoma. Br J Haematol. 2004;124:717–26. 10.1111/j.1365-2141.2004.04834.x 15009059

[9] Ozsahin M, Tsang RW, Poortmans P, Belkacemi Y, Bolla M, Dincbas FO, Landmann C, Castelain B, Buijsen J, Curschmann J, Kadish SP, Kowalczyk A, Anacak Y, Hammer J, Nguyen TD, Studer G, Cooper R, Sengoz M, Scandolaro L, Zouhair A. Outcomes and patterns of failure in solitary plasmacytoma: a multicenter Rare Cancer Network study of 258 patients. Int J Radiat Oncol Biol Phys. 2006;64:210–7. 10.1016/j.ijrobp.2005.06.039 16229966

[10] Wong ET, Lu XQ, Devulapalli J, Mahadevan A. Cyberknife radiosurgery for basal skull plasmacytoma. J Neuroimaging. 2006;16:361–3. 10.1111/j.1552-6569.2006.00062.x 17032388

[11] Theiler G, Schwetz V, Gstettner C, Wowra B, Fürweger C, Steiner J, Schmidt HH. Cyberknife radiosurgery leading to long-lasting complete remission in locally relapsed solitary plasmacytoma of the bone. Ann Hematol. 2015;94:1251–3. 10.1007/s00277-015-2365-4 25859984

[12] Peker S, Abacioglu U, Bayrakli F, Kilic T, Pamir MN. Gamma knife radiosurgery for cavernous sinus plasmacytoma in a patient with breast cancer history. Surg Neurol. 2005;63:174–6. discussion 176–7. 10.1016/j.surneu.2003.12.013 15680666

[13] Alafaci C, Grasso G, Conti A, Caffo M, Salpietro FM, Tomasello F. Cyberknife radiosurgery for cranial plasma cell tumor. Turk Neurosurg. 2014;24:272–5. 10.5137/1019-5149.JTN.7049-12.0 24831374

[14] Chang UK, Lee DH, Kim MS. Stereotactic radiosurgery for primary malignant spinal tumors. Neurol Res. 2014;36:597–606. 10.1179/1743132814Y.0000000381 24773479

